# Multi-omics profiling identifies ADAM9 as a key efferocytosis driver in lung adenocarcinoma

**DOI:** 10.3389/fimmu.2026.1772167

**Published:** 2026-05-25

**Authors:** Guofu Lin, Lanlan Lin, Jianming Zhao, Gongping Chen

**Affiliations:** 1 Department of Pulmonary and Critical Care Medicine, The First Affiliated Hospital, Fujian Medical University, Fuzhou, China; 2 Respiratory Disease Research Institute, the First Affiliated Hospital, Fujian Medical University, Fuzhou, China; 3 The School of Medical Technology and Engineering, Fujian Medical University, Fuzhou, China

**Keywords:** ADAM9, efferocytosis, lung adenocarcinoma, M2 macrophage, single-cell RNA sequencing analysis

## Abstract

**Objective:**

Lung adenocarcinoma (LUAD) remains a leading cause of cancer-related mortality, necessitating identification of novel biomarkers for precision therapy. Herein, we aimed to investigate the role of efferocytosis-related genes (ERGs) in LUAD progression, focusing on ADAM9 as a potential prognostic and therapeutic target.

**Methods:**

TCGA-LUAD datasets including 517 tumor and 59 normal tissues were applied to perform consensus clustering, LASSO regression, and immune infiltration algorithms. A prognostic model was constructed and validated via survival analysis and nomogram. Single-cell sequencing was performed to analyze the distribution of ADAM9 in tumor microenvironment. Additionally, *in vitro* experiments were carried out to analyze the biological function of ADAM9 in LUAD progression.

**Results:**

Eleven ERG signatures, including ADAM9, stratified patients into high- and low-risk groups with significant survival differences. A nomogram integrating ADAM9 and clinical features demonstrated robust predictive accuracy. High ADAM9 expression correlated with immunosuppressive microenvironments, characterized by increased M2 macrophages, neutrophils. Single−cell analysis identified predominant enrichment of ADAM9 in M2 macrophage subsets. Pseudotime trajectory analysis linked ADAM9 expression to M2 macrophage differentiation, while cell−cell interaction studies revealed ADAM9−high M2 macrophages as key signaling hubs in the tumor microenvironment. *In vitro* experiments demonstrated that ADAM9 silencing regulated efferocytosis and polarization in M2 macrophages, and subsequently suppressed tumor cell proliferation and migration via the IL-6/STAT3 signaling pathway.

**Conclusion:**

Our study innovatively constructed a robust prognostic model based on ERG signatures. ERG signatures including ADAM9 not only precisely forecasted the survival outcomes and therapeutic responses of LUAD patients but also comprehensively uncovered the oncogenic role of ADAM9 in promoting tumor progression through multi-dimensional analysis.

## Introduction

Lung cancer remains the leading cause of cancer-related mortality worldwide, accounting for over 1.8 million deaths annually (1). Lung adenocarcinoma (LUAD), the most common histological subtype of non-small cell lung cancer (NSCLC), accounts for 40-50% of all lung cancer cases and 1.2 million annual deaths globally (2). Despite therapeutic advances in targeted therapies and immunotherapy, the 5-year survival rate for metastatic LUAD remains below 15% (3). Early diagnosis is challenging due to nonspecific symptoms, and conventional staging systems often fail to capture tumor heterogeneity or predict treatment response (4). Molecular biomarkers that reflect tumor-immune interactions are urgently needed to optimize precision medicine strategies for this aggressive disease.

Efferocytosis, the phagocytic clearance of apoptotic cells by macrophages, dendritic cells, or tumor cells, plays a critical role in maintaining tissue homeostasis and shaping immune responses (5). In the tumor microenvironment (TME), efferocytosis may exhibit spatiotemporal plasticity: early-stage tumors exploit efferocytosis to suppress inflammation and evade immunosurveillance by releasing “don’t-eat-me” signals (e.g., CD47) and recruiting immunosuppressive macrophages (6). Conversely, in advanced tumors, efferocytosis-mediated antigen presentation by dendritic cells can trigger antitumor T cell responses (7). This duality underscores the need to characterize efferocytosis-related genes (ERGs) as potential prognostic and therapeutic targets in lung cancer.

ADAM9 (A disintegrin and metalloproteinase domain 9), a transmembrane protease, has emerged as an essential player in cancer progression through regulation of cell adhesion, migration, and immune modulation (8–11). Recent studies have demonstrated that ADAM9 exerts an oncogenic role in immune modulation and checkpoints within the tumor microenvironment in hepatocellular carcinoma (HCC) (12). Moreover, ADAM9 promoted the formation of an immunosuppressive tumor microenvironment by reducing the cytotoxic function of CD8+ T cells, thereby contributing to poor prognosis in patients (13), while the role of ADAM9 in efferocytosis-driven immune evasion remains unexplored.

Herein, we present a novel prognostic model based on ADAM9 and other ERGs in LUAD. We identified an eleven-gene signature that stratifies patients into high- and low-risk groups with significant survival differences via TCGA transcriptomic data. High expression of ADAM9 exhibited increased infiltration of immunosuppressive cells. Single−cell analysis further identified predominant enrichment of ADAM9 in M2 macrophage subsets. Pseudotime trajectory analysis indicated that ADAM9 expression correlated dynamically with the differentiation trajectory of macrophages during polarization. *In vitro* experiments validated that ADAM9 knockdown regulated M2 macrophage efferocytosis and polarization via the IL-6/STAT3 signaling pathway. Collectively, our findings establish ADAM9-driven efferocytosis as a critical determinant of immune evasion in lung cancer and provide a translational framework for personalized treatment strategies.

## Materials and methods

### Acquisition of TCGA-LUAD data

The Cancer Genome Atlas (TCGA) (https://portal.gdc.cancer.gov/) has collected and analyzed genomic, transcriptomic, epigenomic, and proteomic data from different types of cancer individuals (14). We utilized the data from TCGA-LUAD cohort, which includes transcriptome data from 517 LUAD tumor patients and 59 normal lung tissue samples. Additionally, clinical data of LUAD patients were downloaded. After a rigorous selection process, we excluded one patient due to missing survival time and tumor stage data, and finally narrowed down the clinical data to a total of 516 LUAD patients with comprehensive clinical information.

### ERGs from GeneCards portal

GeneCards (https://www.genecards.org/) serves as a comprehensive portal website and database (15), offering an abundance of information on over 155,000 human genes, which provides in-depth details on gene expression, function, protein domains, and interactions (16, 17). To identify genes associated with efferocytosis, we utilized a specific approach. Firstly, we conducted a keyword search using “efferocytosis” to identify ERGs in GeneCards. From the resulting list, we selected a total of 157 genes associated with efferocytosis. Finally, we narrowed down our selection to 56 genes (referred to as ERGs) that demonstrated correlation scores greater than 0.4 in the database, applying cutoff of GeneCards Relevance Score > 0.4 and excluding pseudogenes and non-coding RNAs.

### Functional enrichment analysis

To further validate the underlying function of ERGs in different subtypes or risk groups, functional enrichment analysis was conducted on the data. Gene Ontology (GO) is a widely-used tool for gene function annotation, specifically in terms of molecular function (MF), biological pathways (BP), and cellular components (CC). Kyoto Encyclopedia of Genes and Genomes (KEGG) Enrichment Analysis provides valuable resources for studying gene functions and obtaining high-level genomic functional information. In order to gain a better understanding of mRNA carcinogenesis, the ClusterProfiler package in R was utilized to analyze the GO function of potential targets and enrich the KEGG pathways. Boxplots were generated using the R software package ggplot2, while heatmaps were generated using the R software package pheatmap.

### Subtypes identification of ERGs

The RNA-sequencing expression profiles (log_2_(TPM + 1) normalized) and corresponding clinical information for LUAD were obtained from the TCGA dataset. Consistency analysis was performed using the ConsensusClusterPlus R package (v1.54.0), with a maximum of 6 clusters and 80% of the total samples randomly selected 100 times. The clustering algorithm used was “hc” with inner linkage set to ‘ward.D2’. The pheatmap R software package (v1.0.12) was utilized to create clustering heatmaps. The gene expression heatmap includes genes with a standard deviation (SD) greater than 0.1, which serves as the cutoff for excluding low-variation genes. If the number of input genes exceeds 1000, it will select the top 25% genes based on SD after sorting, with a silhouette score greater than 0.5.

### Establishment and validation of the prognostic signature

We extracted data in TPM format from the LUAD dataset and subsequently normalized it using log_2_(TPM + 1). Subsequently, samples that had both RNA-seq data and clinical information (n=516) were randomly divided into training set (70%, n=361) and validation set (30%, n=155) (seed=123) for overfitting control. The log-rank test was employed to compare differences in survival among these groups, with *P* < 0.05 as the significant cutoff. The timeROC (v0.4) analysis was conducted to compare the predictive accuracy of 56 ERGs and the risk score, with area under the curve (AUC) > 0.7 as the cutoff for good predictive performance. Feature selection utilized the least absolute shrinkage and selection operator (LASSO) regression algorithm, employing 10-fold cross-validation, with the R package glmnet utilized for analysis. The construction of a prognostic model involved multivariate Cox regression analysis, with the R package survival used for the analysis. Initially, the data underwent multi-factor Cox regression analysis, followed by iterative steps performed using the step function. Lastly, the final model was selected as the optimal model. Kaplan-Meier curves, *P*-values, and hazard ratios (HR) with a 95% confidence interval (CI) were generated through log-rank tests and univariate Cox proportional hazards regression.

### Nomogram construction

RNA-sequencing expression (level 3) profiles and corresponding clinical information for LUAD were downloaded from the TCGA dataset (n=516). Univariate and multivariate Cox regression analysis were performed to identify the proper terms to build the nomogram, with a significance cutoff of *P* < 0.1 for univariate analysis and *P* < 0.05 for multivariate analysis. The forest was used to show the *P* value, HR and 95% CI of each variable through ‘forestplot’ R package. A nomogram was developed based on the results of multivariate cox proportional hazards analysis to predict the 3-year overall survival. The nomogram provided a graphical representation of the factors which can be used to calculate the risk of recurrence for an individual patient by the points associated with each risk factor through ‘rms’ R package.

### Immune infiltration analysis

For the immune-related analysis, we determined the infiltration scores of 24 immune cell types using the ImmuneCellAI database based on TCGA LUAD dataset, and visualized the correlations of the risk score and candidate 11 ERGs with the degree of immune cell infiltration using the R package ‘ggClusterNet’, with a significance cutoff of *P* < 0.05 and |r| > 0.2. Moreover, the relationship between the ADAM9 expression and immune cell infiltration was analyzed using TIMER and CIBERSORT, with 1000 permutations and requiring *P* < 0.05 for valid samples. Consequently, we assessed the relationship of ADAM9 expression with immune checkpoints, including SIGLEC15, TIGIT, CD274, HAVCR2, PDCD1, CTLA4, LAG3 and PDCD1LG2.

### ScRNA-seq data processing and analysis

Single-cell analysis was performed to validate the expression and distribution of 11 ERGs in patients with LUAD. For the GSE127465 dataset, the Seurat R package (v4.5) was used for quality control, normalization, sample integration, batch effect correction, and cell clustering. Cell clusters were annotated by combining information from the CellMarker database, the SingleR package, and relevant literature. Differential gene expression analysis, slingshot plot analysis, and cell-cell interaction analysis were conducted using R packages.

### Clinical tissue samples

Sixteen pairs of human primary LUAD samples and adjacent lung tissues were collected immediately after surgical resection at The First Affiliated Hospital of Fujian Medical University. All tissue samples were confirmed by histological and pathological examination. None of the patients had received any preoperative chemotherapy or radiotherapy. This study was approved by the Ethics Commission (approval No. 2024-408) and all participants signed the written informed consent.

### Cell lines and cell culture

The human LUAD cell lines (A549, H1975, H460, H1299, SPCA-1), bronchial epithelial cells (BEAS-2B), and THP-1 were purchased from the American Type Culture Collection (ATCC; Manassas, VA, USA). THP-1 cells were stimulated with PMA (100 ng/mL) for 24 h to differentiate monocytes into M2 macrophages. To establish M2 polarization of macrophages in PMA-treated THP-1 cells, IL-4 (20 ng/mL) + IL-13 (20 ng/mL) were used for stimulation for 48 h. Cells were cultured in RPMI-1640 medium, supplemented with 10% fetal bovine serum (FBS, Gibco; Thermo Fisher Scientific, Inc.), and maintained in an incubator at 37 °C with 5% CO_2_. Cell culture dishes and conical-bottom centrifuge tubes were purchased from Bioland (China).

### Western blot

Total protein was extracted from cultured cells using RIPA lysis buffer. Protein samples were separated by SDS-PAGE and transferred onto PVDF membranes. After blocking with 5% non-fat milk, the membranes were incubated overnight at 4°C with primary antibodies against ADAM9 (A03074-1, Boster), IL-6 (GTX110527, GeneTex), STAT3 (ab68153, Abcam), p-STAT3 (ab76315, Abcam), and GAPDH (ab8245, Abcam). Following incubation with HRP-conjugated secondary antibodies, protein bands were visualized using an ECL detection system. GAPDH was used as the loading control.

### Immunohistochemistry

Primary tumors were fixed with 4% paraformaldehyde and embedded in paraffin. The deparaffinized and rehydrated tissue specimens were subjected to antigen retrieval in pH 6.0 citrate buffer for 10 min. Then, the endogenous peroxide activity was blocked with 3% hydrogen peroxide. The sections were incubated with the ADAM9 primary antibodies (Cat. # A03074-1, 1:1000; Boster) overnight at 4°C, and the horseradish peroxidase (HRP) -labeled secondary antibody for 30 min at room temperature. After stained with peroxidase substrate DAB, the slices were observed by Nikon microscope. Images were acquired, and the positive areas were analyzed by using Image J.

### Real-time quantitative PCR (RT-qPCR)

RT-qPCR was carried out following standard protocols (18). Total RNA was extracted from cultured cells and LUAD specimens using Trizol. Subsequently, the RNA was reverse transcribed into cDNA using the PrimeScript™ RT reagent Kit, followed by quantitative PCR using the TB Green Premix Ex Taq II (Takara, Japan). The primers used in this study are listed in **Supplementary Table 1**.

### CFSE-based efferocytosis assay by flow cytometry

To directly assess efferocytosis, we performed CFSE-labeled apoptotic tumor cell uptake assays as previously described (19). Briefly, A549 and H1975 cells were labeled with 5 μM CFSE and induced to undergo apoptosis by 1 μM staurosporine treatment for 4 h. CFSE-labeled apoptotic cells were then co-cultured with M2 macrophages at a 4:1 ratio for 3 h. After co-culture, cells were stained with PE-conjugated anti-CD163 antibody, and the percentage of CD163^+^ CFSE^+^ double-positive cells was quantified by flow cytometry.

### Adenosine 5’-triphosphate detection assay

Cellular ATP levels were detected with ATP detection kit (Beyotime, S0026). Tumor cells were collected and lysed using lysis buffer. The supernatant was obtained by centrifugation at 12,000×g for 6 minutes, after which ATP detection reagent was added. Following a 3-minute incubation, both test samples and standard samples were separately loaded into assay wells. Luminescence was measured using a luminometer (Promega, Madison, WI, USA).

### Cell transfection

Three pre-designed short interfering RNA (siRNA) sequences, which target different coding sequence regions of ADAM9, were directly synthesized (Ribo Bio Tech, Shanghai, China). The sequences of the three ADAM9 siRNA were listed in **Supplementary Table 2**. Macrophages were transiently transfected with siRNA or si-NC using Lipofectamine 3000 (Invitrogen). After 48 h of transfection, the cells were collected for further experiments.

### EdU assay

Cell proliferation was assessed using EdU assays. For EdU assay, cells were stimulated and cultured in a 6-well plate (Bioland, China) for 24 h. The culture medium was then replaced with fresh medium containing EdU (1:1000) and incubated for 2 h. After washing with PBS to remove excess EdU, the cells were fixed with 4% paraformaldehyde for 15 min and treated with 0.3% Triton X-100 for 30 min. Subsequently, the click reaction solution was added to cells for 30 min, following which the nuclei were stained with Hoechst for 10 min. The results were visualized and captured using fluorescence microscopy.

### Transwell assay

Cell migration was assessed using a transwell assay. Cells were pre-cultured in serum-free medium for 48 h. Then, 2.0×10^4^ cells were seeded in the upper chamber of a transwell insert (NEST Biotechnology, Wuxi, China), while the lower chamber was filled with RPMI-1640 medium containing 10% FBS (Bioland, China). After incubating for 48 h, the non-migrating cells on the upper surface of the insert were gently removed using a cotton swab. The migratory cells that had passed through the insert were fixed with 3.7% formalin for 2 min, followed by 100% methanol for 20 minutes at room temperature. These migratory cells were then stained with 0.1% crystal violet for 20 minutes and subsequently visualized under a fluorescence microscope.

### ELISA

The supernatants of macrophage cells after intervention were collected, and IL-6 expression was detected using the Human IL-6 ELISA Kit (SEKH-0013, Solarbio, Beijing, China) following the manufacturer’s instructions. Briefly, 50 μL of supernatant was first added to each well and incubated at room temperature for 2 hours. Biotinylated antibody was then added to each well, with incubation continued for 1 hour. Next, horseradish peroxidase was added to the wells and incubated for an additional 30 minutes. Following this, substrate reagent was added to each well, and absorbance at 450 nm was immediately measured using a microplate reader (iMark Microplate Reader, BIO-RAD, China).

### Statistical analysis

Data analysis was performed using SPSS version 22.0 (IBM Corp.) and GraphPad Prism 8 (GraphPad Software, Inc.) statistical software packages. ImageJ (v1.80, National Institutes of Health) was utilized for semi-quantitative analysis. Each experiment was independently repeated at least three times. The results were expressed as mean ± standard deviation. Statistical differences were calculated using Student’s t-test or one-way analysis of variance, followed by a Tukey’s *post hoc* test. A *P*-value of less than 0.05 was considered statistically significant.

## Result

### Consensus clustering analysis revealed differential gene expression in LUAD samples

**Figure 1:**
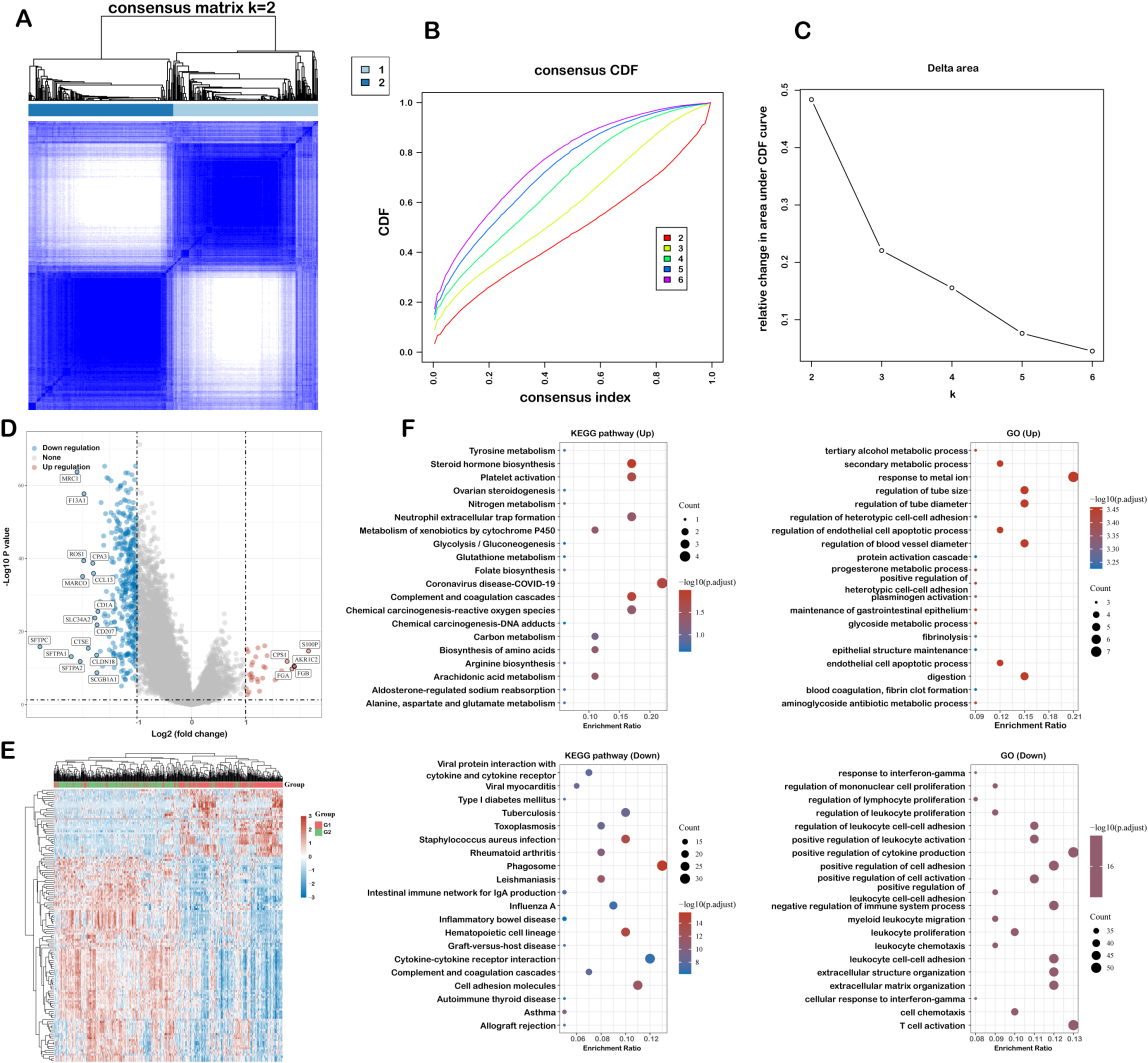
Consensus clustering analysis revealed differential gene expression and functional enrichment analysis in LUAD samples. **(A)** Consensus clustering matrix at k = 2 illustrating sample-similarity patterns in LUAD samples. **(B)** Consensus cumulative distribution function (CDF) curves for k values ranging from 2 to 6. **(C)** The relative change in CDF curve area (Delta area) for k = 2–6 was calculated. **(D)** Volcano plot displaying differentially expressed genes in LUAD samples. **(E)** Heatmap of gene expression patterns between two clusters. **(F)** KEGG and GO enrichment analyses revealed that down-regulated genes were enriched in immune-related pathways (phagosome, interferon-gamma response), supporting the involvement of efferocytosis in LUAD subtype differentiation.

To identify intrinsic molecular subtypes of LUAD and uncover potential gene expression patterns, we performed consensus clustering analysis. A consensus clustering approach was utilized to analyze the LUAD samples, focusing on their intrinsic characteristics. The consensus matrix at k = 2 depicted sample similarity patterns, suggesting two distinct sample clusters (**Figure 1A**). The consensus cumulative distribution function (CDF) curves for k values from 2 to 6, with average values between 0.2 and 0.8, were instrumental in evaluating the stability of the clustering, particularly for the k = 2 case. Additionally, the relative change in the area under the CDF curve (delta area) for k from 2 to 6 was calculated. Its downward-sloping trend indicated that the improvement in the separation between consecutive CDFs decreased as k increased, providing a quantitative measure of the distinctiveness of the two-cluster solution at k = 2 (**Figures 1B, C**). To visualize the transcriptomic differences between these two clusters, a volcano plot was applied to visualize differentially expressed genes (**Figure 1D**). The heatmap in **Figure 1E** displayed the expression patterns of genes. The visual representation allows for the identification of gene clusters with similar expression profiles across different samples. Moreover, KEGG pathway enrichment analysis for up-regulated genes identified steroid hormone biosynthesis, platelet activation, and neutrophil extracellular trap formation as prominent pathways. For down-regulated genes, phagosome, intestinal immune network for IgA production, and influenza pathways are key. In the GO enrichment analysis, up-regulated genes are mainly enriched in tertiary alcohol metabolic, secondary metabolic, and tube-size regulation functions. Down-regulated genes are significantly associated with interferon-gamma response and immune-cell proliferation (**Figure 1F**). Collectively, these analyses suggest that the identified differentially expressed genes are involved in immune modulation networks.

### Prognostic value of efferocytosis-related genes in LUAD

**Figure 2:**
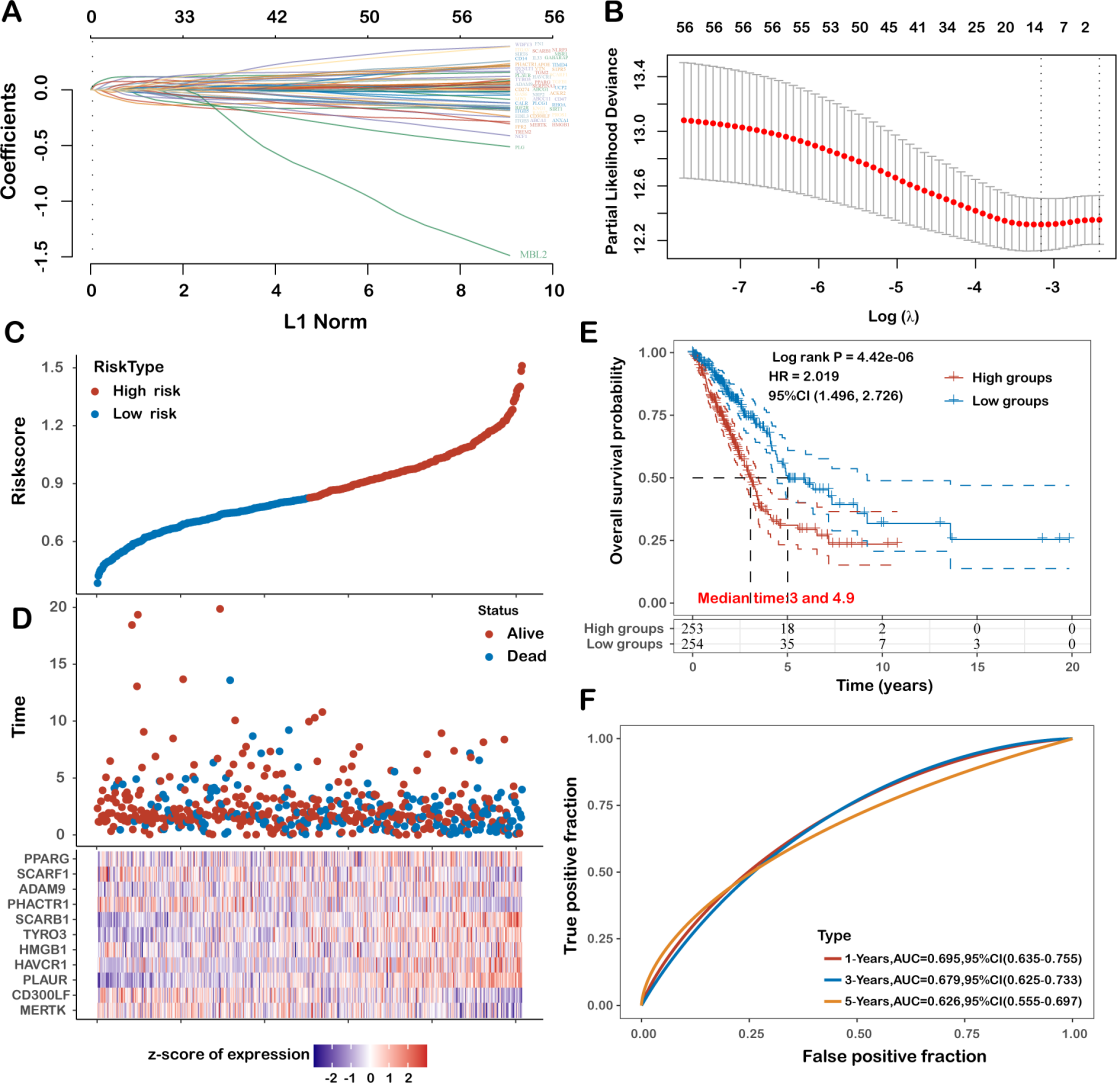
Efferocytosis gene risk model for prognosis in LUAD. **(A, B)** LASSO regression and cross-validation identified optimal ERGs via coefficient shrinkage and λ selection. **(C, D)** Risk score distribution and survival status show that high-risk patients have more deaths and elevated ADAM9 expression. **(E)** Kaplan-Meier analysis confirms that high-risk patients have significantly worse overall survival (HR = 2.019, *P* < 0.001). **(F)** ROC curves assessing the predictive efficacy of the ERGs model for 1-year, 3-year, and 5-year overall survival.

To explore the prognostic value of ERGs in LUAD, LASSO regression analysis was performed. As presented in **Figure 2A**, with the increase of the L1 norm, regression coefficients of ERGs gradually shrank, eventually identifying the optimal gene set. Combined with the cross-validation in **Figure 2B**, the optimal λ value was determined based on the minimum partial likelihood deviance, facilitating the construction of a prognostic risk model.

To visualize the distribution and predictive performance of this risk model, risk score distribution showed that high-risk patients showed a higher proportion of death cases, with significantly elevated risk scores compared to low-risk patients. The heatmap further illustrated distinct expression patterns of key ERGs (e.g., PPARG, SCARF1, ADAM9), highlighting notable inter-group differences (**Figures 2C, D**). Kaplan-Meier survival analysis demonstrated that the high-risk group had poorer OS (HR = 2.019, Log-rank *P* = 4.42×10^-6^), with median survival times of 3 years and 4.9 years, respectively (**Figure 2E**). Additionally, ROC curve analysis was performed to validate the model’s predictive performance. The 1-year, 3-year, and 5-year AUC values were 0.695, 0.679, and 0.626, respectively, indicating the efferocytosis-related gene risk model has considerable predictive potential for LUAD prognosis (**Figure 2F**).

**Figure 3:**
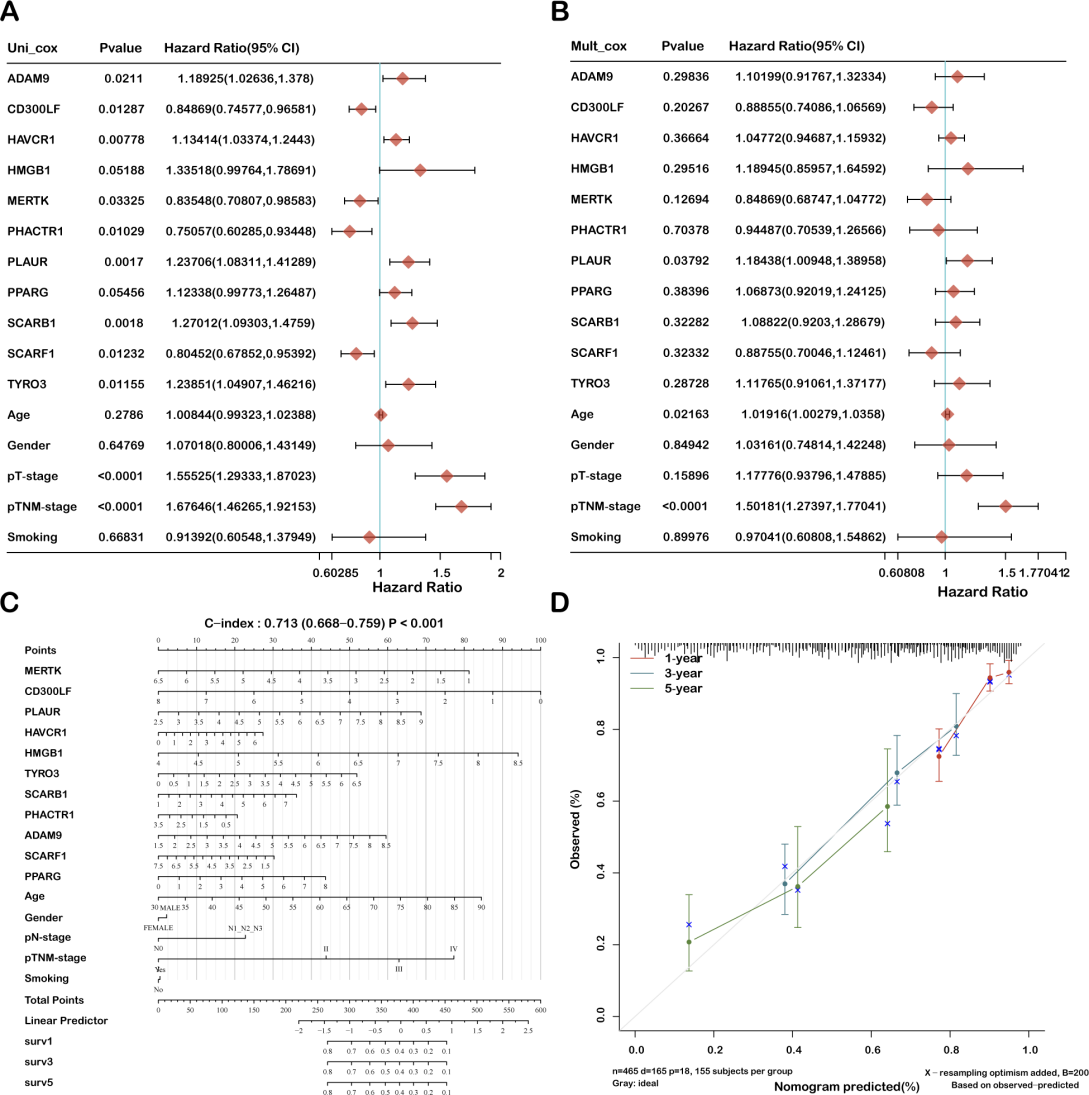
Prognostic analysis of efferocytosis-related genes and construction of a nomogram in LUAD. **(A, B)** Univariate and multivariate Cox regression identified ADAM9 and other ERGs as independent prognostic factors. **(C)** A nomogram was constructed based on multiple influencing factors in LUAD. **(D)** Calibration curves assessing consistency between nomogram-predicted and observed 1−year, 3−year, and 5−year survival probabilities in LUAD.

To identify independent prognostic factors and develop a clinically applicable prediction tool, univariate Cox regression analysis was initially employed to screen prognosis-associated variables. Results revealed that multiple ERGs (e.g., MERTK, PLAUR, ADAM9) and clinical features (age, TNM stage, etc.) showed significant associations with prognosis (**Figure 3A**). Subsequently, multivariate Cox regression analysis was performed, identifying several independent prognostic factors, including specific ERGs and clinical parameters (**Figure 3B**). Based on these independent factors, a nomogram was further constructed. The nomogram exhibited a C-index of 0.713 (95% CI: 0.668-0.759, *P* < 0.001), indicating excellent predictive accuracy for LUAD prognosis (**Figure 3C**). Additionally, calibration curves were used to evaluate the consistency between predicted and actual survival outcomes (**Figure 3D**). The results demonstrated favorable agreement at 1-year, 3-year, and 5-year time points, confirming the nomogram’s reliability in predicting the survival outcomes of LUAD patients by integrating ERGs and clinical variables.

### External validation of the efferocytosis-related gene prognostic model

To validate the robustness of our 11-gene prognostic model, we applied the same risk scoring formula to the independent GSE31210 LUAD cohort (n = 226). Patients were stratified into high- and low-risk groups using the median risk score threshold from the TCGA training cohort.

As shown in **Supplementary Figure 1A**, the risk score distribution and patient survival status in GSE31210 closely recapitulated the TCGA patterns, with mortality concentrated in the high-risk group. Kaplan-Meier analysis confirmed that high-risk patients had significantly poorer overall survival (HR = 2.387, log-rank *P* = 9.82×10^-6^; **Supplementary Figure 1B**). The **Supplementary Figure 1C** illustrated the expression patterns of the 11 ERGs across the GSE31210 cohort, revealing distinct expression profiles between high- and low-risk groups that were consistent with our TCGA observations. Time-dependent ROC analysis yielded AUC values of 0.689, 0.636, and 0.570 for 1-year, 3-year, and 5-year survival, respectively, demonstrating stable predictive performance (**Supplementary Figure 1D**).

Collectively, these external validation results demonstrated the robustness and generalizability of our 11-gene efferocytosis-related prognostic model in stratifying LUAD patients by survival outcomes.

### ADAM9 mediated immune cell crosstalk and M2 polarization in LUAD

**Figure 4:**
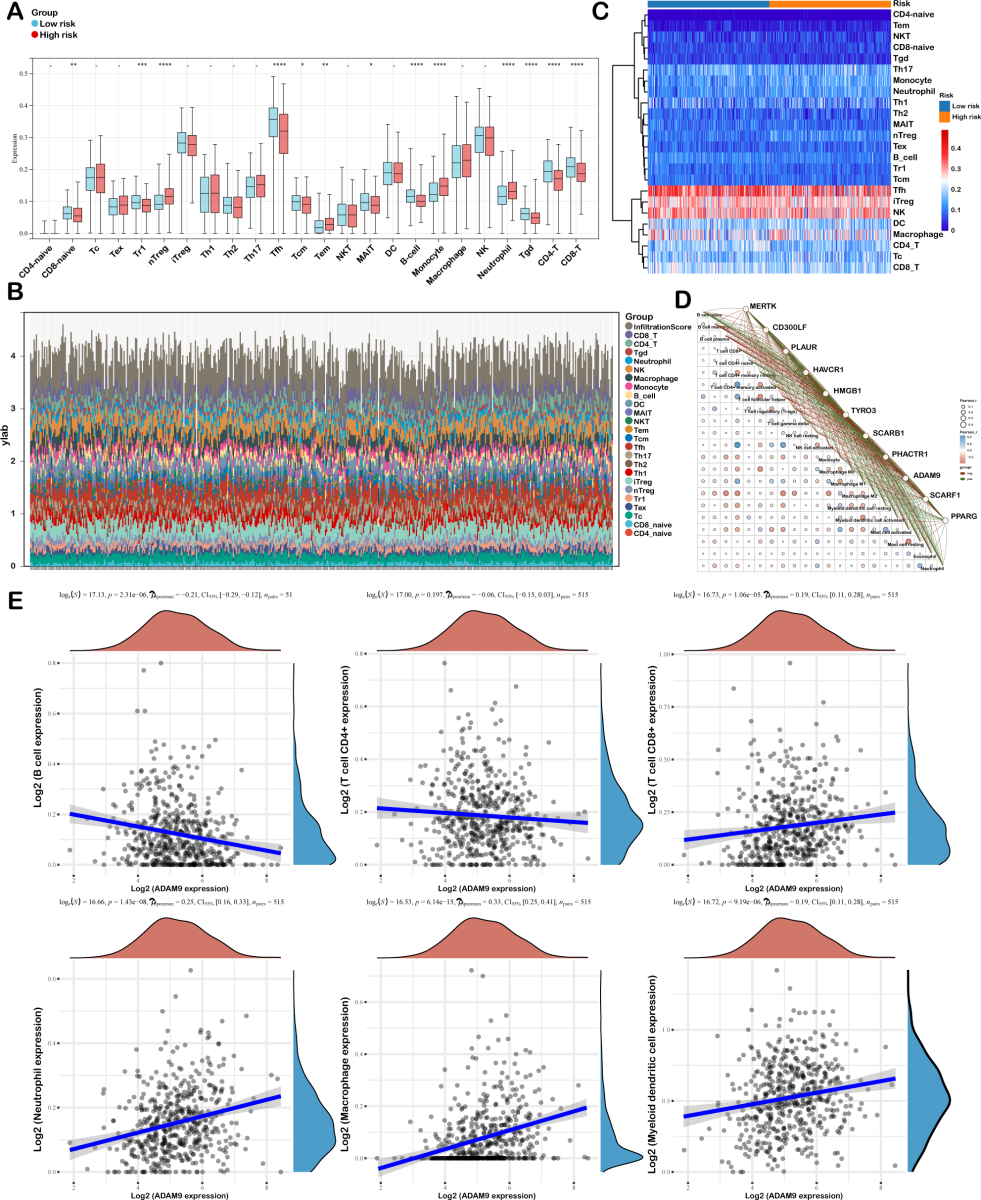
ADAM9 expression correlates with immune cell infiltration in LUAD. **(A)** The expression discrepancies of designated genes between ADAM9-high and ADAM9-low groups. **(B)** The compositional distribution of immune cell in LUAD. **(C)** The heatmap visualizes immune infiltration levels of diverse immune cell types between the high- and low-expression group of ADAM9. **(D)** Interaction relationships between ERGs and immune cells. **(E)** Correlations between ADAM9 expression and the infiltration levels of immune cell subsets.

Given that ADAM9 was identified as a key ERG, we next sought to elucidate its immunological role in the LUAD microenvironment. **Figure 4A** showed significant disparities in immune cell infiltration levels between ADAM9 high- and low-expression groups, indicating that ADAM9 expression profoundly influences immune cell recruitment within the LUAD microenvironment. We further analyzed the proportional distribution of immune cell infiltration across LUAD datasets, and the result uncovered heterogeneous immunological profiles inherent to the tumor microenvironment (**Figure 4B**). Quantitative evaluation in **Figure 4C** showed distinct immune cell infiltration scores between high- risk and low-risk groups, highlighting notable differences in immune microenvironment features associated with ADAM9 expression. Meanwhile, we explored the correlation between ERGs and immune cells, revealing potential regulatory networks linking ADAM9 to immune modulation (**Figure 4D**). **Figure 4E** presented that specific immune cell subsets exhibited positive or negative correlations with ADAM9 expression. Notably, ADAM9 exhibited the strongest correlation with macrophages (ρ = 0.33), underscoring its pivotal role in regulating macrophage infiltration. Collectively, these results establish ADAM9 as a key regulator of immune cell dynamics and immune-related gene interactions in LUAD, providing insights into its immunopathological mechanisms.

**Figure 5:**
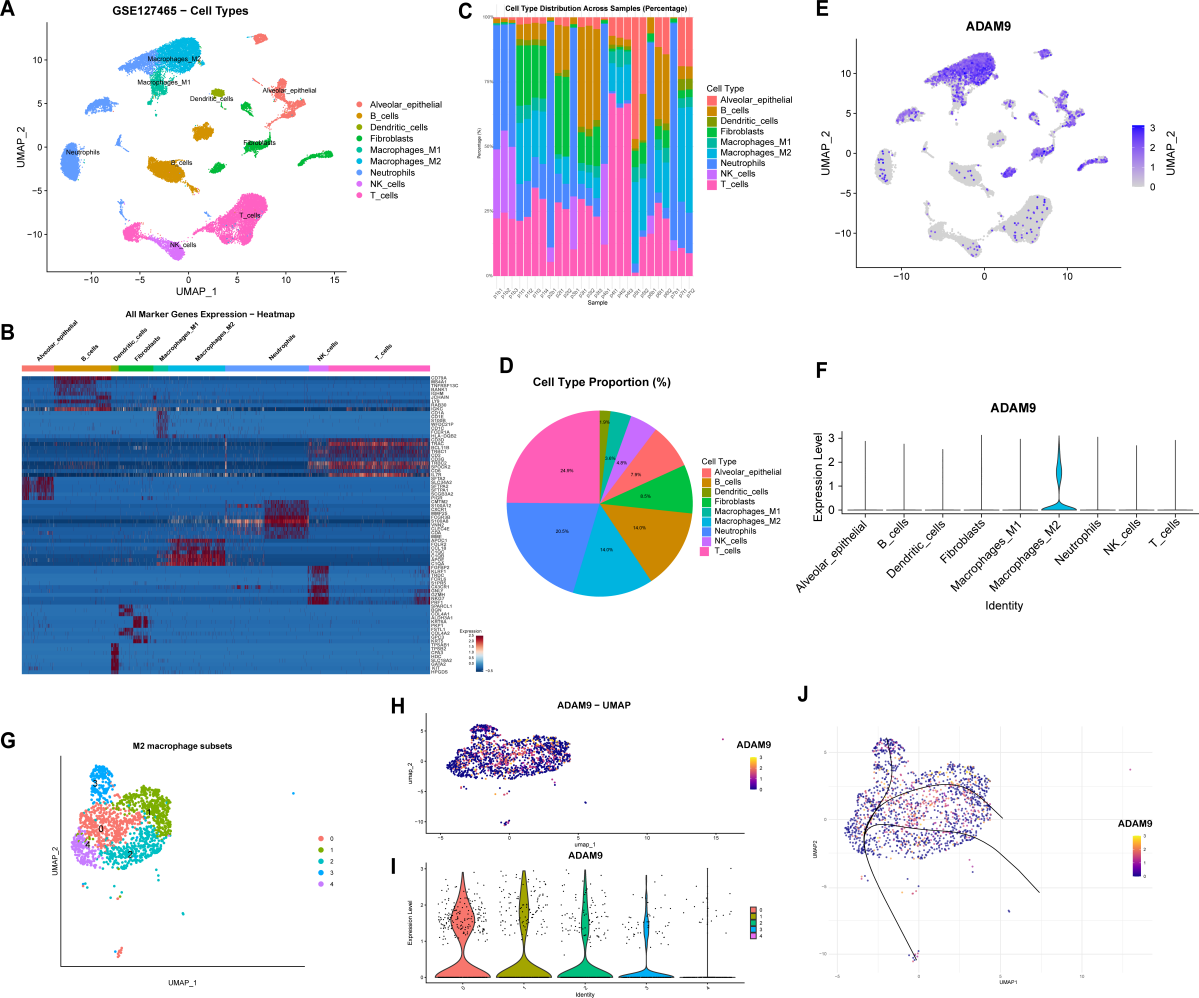
Single-cell RNA-seq revealed ADAM9 enrichment in M2 macrophages. **(A)** Single-cell clustering analysis of NSCLC (GSE127485) depicting diverse cell type. **(B)** The expression levels of marker genes across various cell types. **(C)** The composition of cell type proportions in different samples. **(D)** The overall percentage of each cell type in the dataset. **(E)** The expression distribution of ADAM9 across distinct cell subsets. **(F)** Violin plot comparing ADAM9 expression levels among different cell types. **(G)** UMAP plot showing clustering results within the M2 macrophage subset. **(H)** The expression distribution of ADAM9 across M2 macrophage subclusters. **(I)** ADAM9 expression levels among different M2 macrophage subclusters. **(J)** Pseudotime trajectory analysis showing ADAM9 expression gradually increased from early (C4/C3) to terminal (C1/C2/C0) M2 subclusters.

To dissect ADAM9’s expression and functional roles at single-cell resolution, we initially analyzed its expression and regulatory roles via scRNA-seq analysis. Single-cell clustering of the GSE127465 dataset identified multiple cell types according to the typical type-specific gene markers in the LUAD (**Figures 5A, B**, **Supplementary Figure 2**), with cell types annotated using canonical lineage markers: SFTPC (alveolar epithelial cells), CD3D (T cells), CD19 (B cells), COL1A1 (fibroblasts), CD86 (M1 macrophages), CD163 (M2 macrophages), CSF3R (neutrophils), CD1C (dendritic cells), and NKG7 (NK cells). In addition, we also analyzed the expression patterns of key efferocytosis genes using UMAP and violin plots, as shown in **Supplementary Figures 3**, **4**, which revealed cell-type-specific expression profiles of these genes across the entire immune and stromal compartments. Cell composition analysis quantified the proportional distribution of each cell type across samples and in the global dataset, providing context for subsequent ADAM9-focused analyses (**Figures 5C, D**), with proportions calculated as the percentage of each cell type relative to the total cell count per sample and globally. Strikingly, ADAM9 expression was heterogeneously distributed across cells (**Figure 5E**), with prominent enrichment in M2 macrophages (**Figure 5F**), as confirmed by violin plot visualization of ADAM9 expression across all annotated cell types. To further resolve this specificity, we subclustered M2 macrophages (**Figure 5G**) using the same dimensionality reduction and clustering pipeline applied to the full dataset, yielding 5 distinct M2 subclusters and found ADAM9 expression varied distinctly across these M2 subclusters (**Figures 5H, I**), with highest expression detected in a subset of M2 subclusters. To investigate whether ADAM9 correlates with M2 differentiation dynamics, slingshot-based pseudotime trajectory analysis revealed that ADAM9 expression gradually increases along M2 macrophage differentiation, from early subclusters (C4/C3) to terminal subclusters (C1/C2/C0), exhibiting a clear low-to-high expression gradient (**Figure 5J**), where pseudotime was inferred with a selected root subcluster, and ADAM9 expression was mapped along the trajectory to reveal its dynamic upregulation during M2 differentiation.

**Figure 6:**
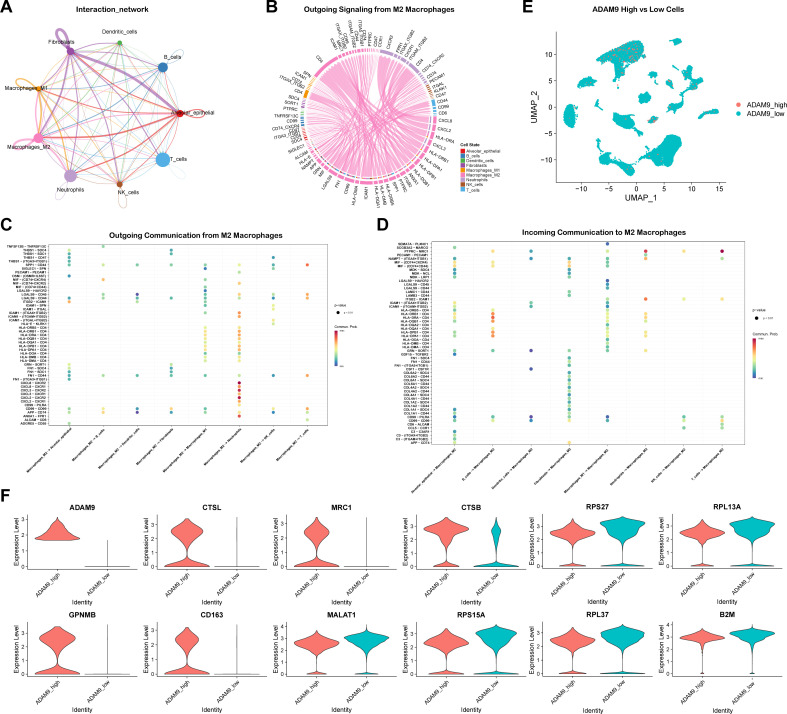
ADAM9-high M2 macrophages exhibited distinct intercellular communication patterns. **(A)** M2 macrophages served as central hubs in cell-cell interaction networks. **(B)** Outgoing signaling network depicting signal pathways from M2 macrophages to other cell types. **(C)** Outgoing communication heatmap of ligand-receptor pair strength from M2 macrophages. **(D)** Incoming communication heatmap of ligand-receptor pair strength received by M2 macrophages. **(E)** UMAP plot showing distribution of cells in the population. **(F)** ADAM9-high M2 macrophages showed elevated expression of efferocytosis-related genes (MRC1, CD163, GPNMB).

To understand how ADAM9-high M2 macrophages communicate with other cell types, signaling cell-cell interaction networks first confirmed M2 macrophages as central mediators of crosstalk with other cell types (**Figure 6A**), while the outgoing signaling network visualized M2-derived signal pathways (**Figure 6B**), highlighting key ligand-receptor pairs involved in M2 macrophage signaling to other cell types. Additionally, ligand-receptor communication analyses quantified the strength of outgoing (from M2) and incoming (to M2) signals, revealing ADAM9-dependent shifts in intercellular signaling (**Figures 6C, D**), with communication strength calculated as the sum of interaction probabilities for each ligand-receptor pair, and statistical testing performed to identify significant differences between ADAM9-high and ADAM9-low groups. To correlate these communication patterns with ADAM9 expression, we defined ADAM9-high and ADAM9-low populations by UMAP (**Figure 6E**), using the median expression of ADAM9 in M2 macrophages as the threshold to stratify cells into high- and low-expression subgroups. Violin plots then verified distinct expression profiles of core efferocytosis genes (including ADAM9) across these populations (**Figure 6F**), confirming that ADAM9-high M2 macrophages exhibit elevated expression of ERGs such as MRC1, CD163, and GPNMB relative to ADAM9-low cells.

Additionally, to assess the broader immune implications of ADAM9 expression, immune-related analyses revealed significant differences between ADAM9-high and ADAM9-low groups. **Supplementary Figures 5A, B** showed differential expression of immune checkpoints between groups, including SIGLEC15, HAVCR2, and PD-L2. Moreover, **Supplementary Figure 5C** demonstrated that ADAM9-high group featured increased infiltration of T regulatory cells, macrophage M0, and activated NK cells. We further presented pronounced differences in infiltration percentages across cell types between ADAM9-high and -low groups (**Supplementary Figure 5D**). Collectively, these results establish ADAM9 as a pivotal regulator of both M2 macrophage differentiation and immune cell communication within the LUAD microenvironment.

### ADAM9 mediated efferocytosis and polarization capacity of macrophage cells

**Figure 7:**
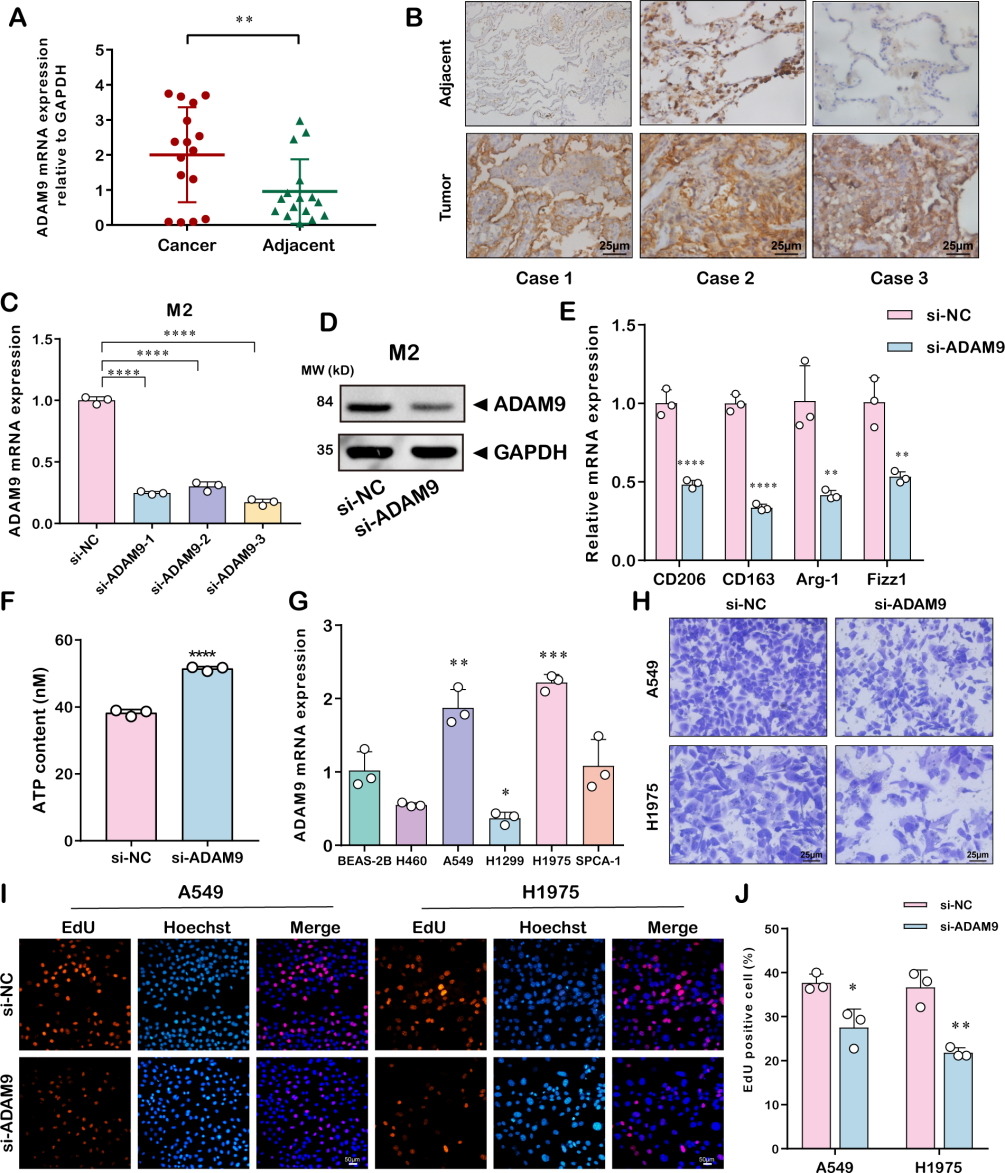
Effects of ADAM9 knockdown in macrophage cells. **(A)** Relative mRNA expression of ADAM9 in LUAD tissues and adjacent tissues. **(B)** ADAM9 expression in LUAD tissues evaluated by IHC. **(C, D)** ADAM9 expression in M2 macrophages transfected with siRNA. **(E)** RT-qPCR analysis of M2 polarization markers in si-ADAM9-transfected M2 macrophages. **(F)** ATP content assay in M2 macrophages transfected with si-ADAM9. **(G)** ADAM9 mRNA expression in normal bronchial epithelial cells and LUAD cell lines. **(H)** Transwell migration assay showing the migratory ability of A549 and H1975 cells co-cultured with M2 macrophages. **(I, J)** EdU assay detecting the proliferative capacity of LUAD cells co-cultured with M2 macrophages. *P < 0.05, **P < 0.01, ***P < 0.001, ****P < 0.0001.

To validate the clinical relevance of ADAM9 expression in LUAD, we initially detected ERGs expression in cancer and corresponding adjacent tissues using three independent batches of clinical samples. As shown in **Figure 7A**, **Supplementary Figure 6**, ADAM9 RNA expression was significantly higher in cancer tissues compared to adjacent tissues. Then we further detected ADAM9 protein expression in 16 LUAD tissues and adjacent tissues with immunohistochemistry, with negative controls confirming staining specificity. Representative IHC images of ADAM9 expression were shown in **Figure 7B**, indicating its potential role in carcinogenesis.

Based on the scRNA-seq finding that ADAM9 was enriched in M2 macrophages,we subsequently investigated the functional role of ADAM9 in M2 macrophages. M2 macrophages were initially induced by activating of monocyte-derived macrophages, followed by transfection with siRNAs to knock down ADAM9 expression. The results demonstrated that ADAM9 expression was significantly downregulated in M2 macrophages transfected with si-ADAM9-1. Accordingly, this siRNA was selected for subsequent experiments (**Figures 7C, D**). To evaluate the effect of ADAM9 silencing on macrophage polarization, we detected the expression alterations of M2 macrophage polarization markers. All experiments were performed in three independent biological replicates. As shown in **Figure 7E**, **Supplementary Figure 7**, the expression of M2 markers was notably decreased, whereas M1 markers were significantly elevated, indicating impaired M2 polarization capacity and concomitant promotion of M1 polarization upon ADAM9 knockdown. To directly assess efferocytosis, we performed CFSE-labeled apoptotic tumor cell uptake assays co-cultured with M2 macrophages. Flow cytometry quantification of CD163^+^ CFSE^+^ double-positive cells demonstrated that ADAM9 knockdown significantly increased the double-positive rate (**Supplementary Figure 8**), indicating enhanced efferocytic capacity of M2 macrophages toward apoptotic tumor cells. Consistently, ATP levels were also significantly elevated upon ADAM9 knockdown (**Figure 7F**). Together, these results provide direct and complementary evidence that ADAM9 knockdown enhances macrophage efferocytosis.

To determine whether ADAM9-dependent macrophage polarization affects LUAD cell behavior, we performed co-culture experiments. Considering ADAM9 as a vital regulatory molecule for macrophage, we next explored whether macrophages affected the proliferation of LUAD cells due to ADAM9-dependent polarization. We first detected the ADAM9 expression of LUAD cell lines to eliminate potential confounding effects from inherent ADAM9 expression. Based on the expression profiles, we selected the A549 and H1975 cell lines for subsequent functional assays (**Figure 7G**). Subsequently, we co-cultured ADAM9-knockdown M2 macrophages with LUAD cells to systematically evaluate the phenotypes of tumor cells. EdU and Transwell assays demonstrated that the proliferative and migrative abilities of LUAD cells were significantly reduced after ADAM9 knockdown, suggesting that M2 macrophages with ADAM9 knockdown effectively attenuated the malignant proliferation and migration capacity of co-cultured tumor cells. (**Figures 7H-J**).

### ADAM9 regulated M2 macrophage polarization via IL-6/STAT3 axis

**Figure 8:**
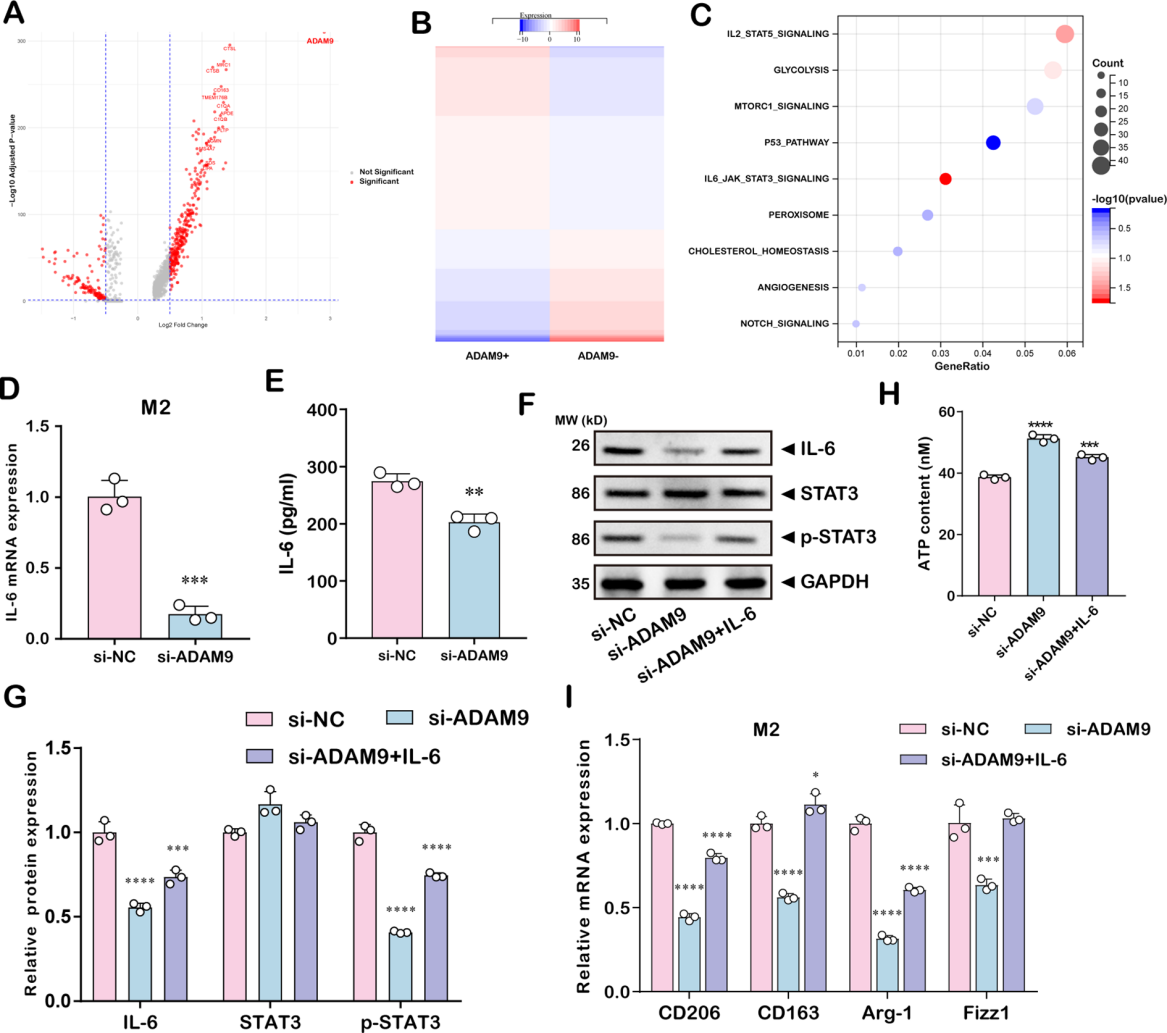
ADAM9 regulated M2 macrophage function via the IL-6/STAT3 axis. **(A, B)** DEGs between ADAM9-high/low cell groups via scRNA-seq. **(C)** KEGG plot showing pathway enrichment of DEGs in ADAM9-high/low clusters. **(D, E)** RT-qPCR and ELISA analysis of IL-6 expression in M2 macrophages transfected with si-ADAM9. **(F, G)** Western blot analysis of IL-6, STAT3, and p-STAT3 protein levels in M2 macrophages. **(H)** ATP content assay in M2 macrophages after si-ADAM9 and IL-6 intervention. **(I)** RT-qPCR analysis of polarization markers in M2 macrophages. *P < 0.05, ***P < 0.001, ****P < 0.0001.

To elucidate the downstream signaling pathways through which ADAM9 regulates M2 macrophage function, we deeply explored the potential pathways by analyzing DEGs between ADAM9-high and ADAM9-low clusters based on scRNA-seq data. The volcano plot identified genes with significant differential expression, while the heatmap showed distinct transcriptional patterns between the two groups (**Figures 8A, B**). Further KEGG enrichment analysis indicated that the DEGs were significantly enriched in IL-6/STAT3 axis signaling pathway (**Figure 8C**). To validate the underlying mechanism in M2 macrophages, we first investigated the expression and secretion of IL-6 in ADAM9-manipulated macrophages with si-NC group as a control. RT-qPCR and ELISA results revealed that IL-6 expression was significantly downregulated in ADAM9-knockdown macrophages (**Figures 8D, E**). To confirm pathway activation status, the activation status of the IL-6/STAT3 signaling pathway was further detected via western blot. The results demonstrated that ADAM9 knockdown contributed to a significant decrease in the protein levels of IL-6 and p-STAT3. Notably, exogenous supplementation of IL-6 reversed the inhibitory effect of ADAM9 depletion on the IL-6/STAT3 pathway (**Figures 8F, G**). To link pathway modulation to M2 macrophage functional phenotypes, we assessed polarization capacity and efferocytosis ability. The results indicated that exogenous IL-6 could reverse the functional alterations induced by ADAM9 knockdown in M2 macrophages (**Figures 8H, I**).

## Discussion

Lung adenocarcinoma remains a significant global health burden, with a dismal 5-year survival rate for metastatic cases. Immune checkpoint inhibitors (ICIs) have shown substantial clinical benefits in terms of both effectiveness and safety, emerging as a beacon of hope for the treatment landscape of LUAD (20, 21). Given the circumstance, the development of effective precision medicine strategies is crucial, and molecular biomarkers that can accurately predict prognosis, immune microenvironmental status and immune response are urgently needed (22). In this study, we focused on ERGs, particularly ADAM9, to develop a novel prognostic model for LUAD, and our findings have important implications.

Our consensus clustering analysis of LUAD samples revealed distinct gene expression patterns, with differential genes significantly involved in immune modulation networks, which aligned with the known role of efferocytosis in shaping the tumor immune microenvironment. The identified pathways, such as steroid hormone biosynthesis, platelet activation, and neutrophil extracellular trap formation for the up-regulated genes, and phagosome, intestinal immune network for IgA production, and influenza pathways for the down-regulated genes, highlighted the complex interplay between tumor cells and the immune system (23). For instance, disrupting the platelet activation pathway might hinder tumor angiogenesis, as platelets can release growth factors that promote blood vessel formation in the tumor microenvironment (24, 25). Similarly, targeting the phagosome pathway could potentially interfere with the tumor’s ability to evade the immune system by modulating the process of apoptotic cell clearance (26, 27). The above pathways may serve as potential therapeutic targets, and further investigation into their roles in LUAD progression could lead to the development of novel treatment strategies.

The construction of a prognostic risk model based on ERGs demonstrated its predictive potential for LUAD prognosis. The LASSO regression analysis effectively identified an optimal gene set, and the resulting risk score could stratify patients into high- and low-risk groups with significant survival differences, which may potentially guide clinical decision-making by identifying high-risk patients who may benefit from more intensive surveillance or adjuvant therapy. The 1-year, 3-year, and 5-year AUC values of the ROC curve also indicated that the model has a certain degree of accuracy in predicting patient outcomes. Moreover, the nomogram constructed based on independent prognostic factors, including specific ERGs and clinical parameters, exhibited excellent predictive accuracy, as evidenced by its C-index and calibration curves. This nomogram provides a practical tool for clinicians to estimate the survival probability of LUAD patients, enabling more personalized treatment decisions. The integration of this risk model with established clinical guidelines could facilitate dynamic risk stratification during patient follow−up, allowing timely adjustment of therapeutic strategies. Notably, ADAM9 emerged as a critical regulator of M2 macrophage polarization and immune crosstalk, suggesting its potential as both a predictive biomarker for immunotherapy response and a therapeutic target.

ADAM9 has emerged as an essential element in LUAD biology. Our immune infiltration analysis showed that ADAM9 expression profoundly influences immune cell recruitment and the immune microenvironment in LUAD. High expression of ADAM9 was associated with increased infiltration of immunosuppressive cells, such as M2 macrophages and neutrophils. Previous studies have reported that macrophages orchestrated efferocytosis through sequential recognition of phosphatidylserine on apoptotic cells and subsequent phagocytic engulfment (28, 29), which is mediated by receptors such as TIM-4/BAI1 and MFG-E8/αvβ3 integrin, as well as bridging molecules (30–32). Concurrently, macrophages establish an immunosuppressive microenvironment via multiple mechanisms, including the secretion of TGF-β1 and IL-10 (33, 34), downregulating CD80/CD86 costimulatory molecules and MHC expression (35, 36), depleting arginine via arginase-1 (37), and inhibiting NLRP3 inflammasome activation through succinate accumulation (38, 39). In addition, neutrophils also play a crucial role in the immune response. Tumor cells might induce the differentiation of CD74+ SiglecF+ neutrophils through the secretion of factors (e.g., IL-8), which may influence anti-tumor immunity by regulating the T cell response (40, 41). Notably, under specific tumor microenvironment conditions, neutrophils can acquire immunosuppressive functions, such as polarization toward an N2-like phenotype that inhibits T cell activation (42, 43). Moreover, neutrophils can be efferocytosed by macrophages, which promotes macrophage polarization towards an M2 phenotype (44). These evidences suggested that ADAM9 may contribute to immune evasion in LUAD, potentially explaining the poor prognosis of patients with high ADAM9 expression. The strong correlation between ADAM9 and macrophages/neutrophils further emphasizes its role in regulating the TME.

Based on the suppressive effect of ADAM9 in the immune microenvironment, *in vitro* experiments were further applied. The results demonstrated that ADAM9 knockdown in macrophage suppressed the proliferation and migration of LUAD cells. Similarly, ADAM9 upregulation enhanced the malignant progression of pancreatic ductal adenocarcinoma (PDAC) by downregulating the expression of adhesion junction proteins, upregulating the WNT signaling pathway, and stabilizing the KRAS protein (45, 46). Evers et al. reported that knockdown of ADAM9 downregulated proliferation related gene sets and mediated pro-proliferative effects in multiple myeloma (47). These findings suggested that targeting ADAM9 could be a potential therapeutic strategy for LUAD. Inhibition of ADAM9 may not only suppress tumor cell growth and metastasis but also modulate the immune microenvironment, enhancing the anti-tumor immune response.

Additionally, our study demonstrated that ADAM9 regulated macrophage efferocytosis and M2 polarization through the IL-6/STAT3 signaling axis. Similarly, Liu et al. demonstrated that ADAM9 promotes IL-6/IL-6R-STAT3 axis signaling in lung cancer, leading to suppression of IL-12p40 secretion and MHC class II expression in dendritic cells and macrophages, thereby creating an immunosuppressive environment (13). Importantly, Campana et al. established the STAT3-IL-6-IL-10 pathway as a positive regulator of macrophage efferocytosis, survival, and phenotypic conversion, directly linking debris engulfment to tissue repair (48). Furthermore, Frisdal et al. showed that IL-6 induces expression of efferocytosis-related molecules including c-mer proto-oncogene tyrosine kinase (MERTK) and transglutaminase 2 in human macrophages via the JAK2/STAT3 signaling pathway, enhancing the capacity to phagocytose apoptotic cells (49). Additionally, Kuo et al. reported that Rab37-mediated exocytosis of IL-6 activates STAT3 signaling in macrophages to promote M2 polarization and lung cancer progression (50). Collectively, these studies corroborate our findings that ADAM9 serves as an upstream regulator sustaining IL-6/STAT3 signaling to promote M2 polarization and modulate efferocytosis, positioning the axis as a potential therapeutic target in LUAD.

However, this study has several limitations. First, the prognostic model and the role of ADAM9 remain to be validated in larger-scale independent cohorts. The current analysis was mainly based on TCGA data and a limited number of clinical samples, and external validation is essential to confirm the generalizability of our findings. Second, although our data suggested that ADAM9 contributed to immune suppression potentially via the IL-6/STAT3 axis, the direct causal link and the specificity of this signaling axis in mediating the effects of ADAM9 on efferocytosis require further validation. Third, the effects of ADAM9 on additional malignant phenotypes of LUAD, such as angiogenesis and epithelial-mesenchymal transition, have yet to be fully explored. Investigating these areas could provide a more comprehensive understanding of the role of ADAM9 in LUAD progression.

## Conclusion

In conclusion, our study established ADAM9-driven efferocytosis as a critical determinant of immune evasion in LUAD and provided a translational framework for personalized treatment strategies. The prognostic model based on ERGs, especially ADAM9, shows promise in predicting patient outcomes. Future research should aim to validate these findings in larger cohorts, clarify the molecular mechanisms, and explore the potential of targeting ADAM9 for the treatment of LUAD.

## Data Availability

The data presented in the study are from public databases, including GEO (accession numbers: GSE127465, GSE31210) and TCGA-LUAD. No original data were generated. The data are already publicly accessible.
