# Supplementary material for: Multi-omics profiling identifies ADAM9 as a key efferocytosis driver in lung adenocarcinoma
Source: Front Immunol. 2026 May 25;17:1772167. doi: 10.3389/fimmu.2026.1772167 (PMC13243381; doi:10.3389/fimmu.2026.1772167)
Supplement: Supplementary file 1 [file DataSheet1.pdf]

Supplementary materials

Multi-omics profiling identifies ADAM9 as a key efferocytosis driver in lung adenocarcinoma

Guofu Lin[#], Lanlan Lin[#], Jianming Zhao[#], Gongping Chen[*]

Supplemantary tables

Table S1 primers sequences

Gene	*Forward (5'-3')*	*Reverse (5'-3')*
ADAM9	TCCATTGCTCTTAGCGACTGT	GGGGTTCAATCCCATAACTCG
CD300LF	CTGGCTCTCAGGCTACTCCAT	CGCCAAATAGCTCCTCGACA
PLAUR	TGTAAGACCAACGGGGATTGC	AGCCAGTCCGATAGCTCAGG
HAVCR1	TGGCAGATTCTGTAGCTGGTT	AGAGAACATGAGCCTCTATTCCA
HMGB1	TATGGCAAAAGCGGACAAGG	CTTCGCAACATCACCAATGGA
PHACTR1	CCTCGGAGGATGATATAGACCG	TGGCAAACTTACTTCTCCTGC
PPARG	GGGATCAGCTCCGTGGATCT	TGCACTTTGGTACTCTTGAAGTT
SCARB1	CCTATCCCCTTCTATCTCTCCG	GGATGTTGGGCATGACGATGT
SCARF1	CCGATCAGACCTCAAGGACAG	CCCAGGGTAGCTTGTGGGA
TYRO3	CAGCCGGTGAAGCTCAACT	TGGCACACCTTCTACCGTGA
MERTK	CTCTGGCGTAGAGCTATCACT	AGGCTGGGTTGGTGAAAACA
CD206	TCCGGGTGCTGTTCTCCTA	CCAGTCTGTTTTTGATGGCACT
CD163	TTCCTGTTCTGGACGTGTGG	AGCTGGACCACAGCCAAGTT
Arg-1	CCTTTGCTGACATCCCTAAT	GATTCTTCCGTTCTTCTTGACT
Fizz1	CCGTCCTCTTGCCTCCTTC	CTTTTGACACTAGCACACGAGA
CD86	CTGCTCATCTATACACGGTTACC	GGAAACGTCGTACAGTTCTGTG
iNOS	GAGCCAGGCCACCTCTATGT	GTCCTCGACCTGCTCCTCAT
TLR2	ATCCTCCAATCAGGCTTCTCT	GGACAGGTCAAGGCTTTTTACA
IL-1B	AAACGAATGAAGTGCTCCTTCAGC	ACCTCGTTGTTCACCACAAGAGGT
GAPDH	CATgTTCGTCATgGGTgTgAACCA	AGTgATgGCATgGACTgTgGTCAT

Table S2 siRNA sequences for ADAM9

Gene	*5'-3'*
si-ADAM9-1	CUCCUUGGAGAUUAACUAGUU
si-ADAM9-2	CCTTCCAGAGTATTGTGACGGCAAA
si-ADAM9-3	CCCGACUCCUUUAUCCUAUGACUUA
si-NC	TGCGCTAGGCCTCGGTTGC

Supplementary figures

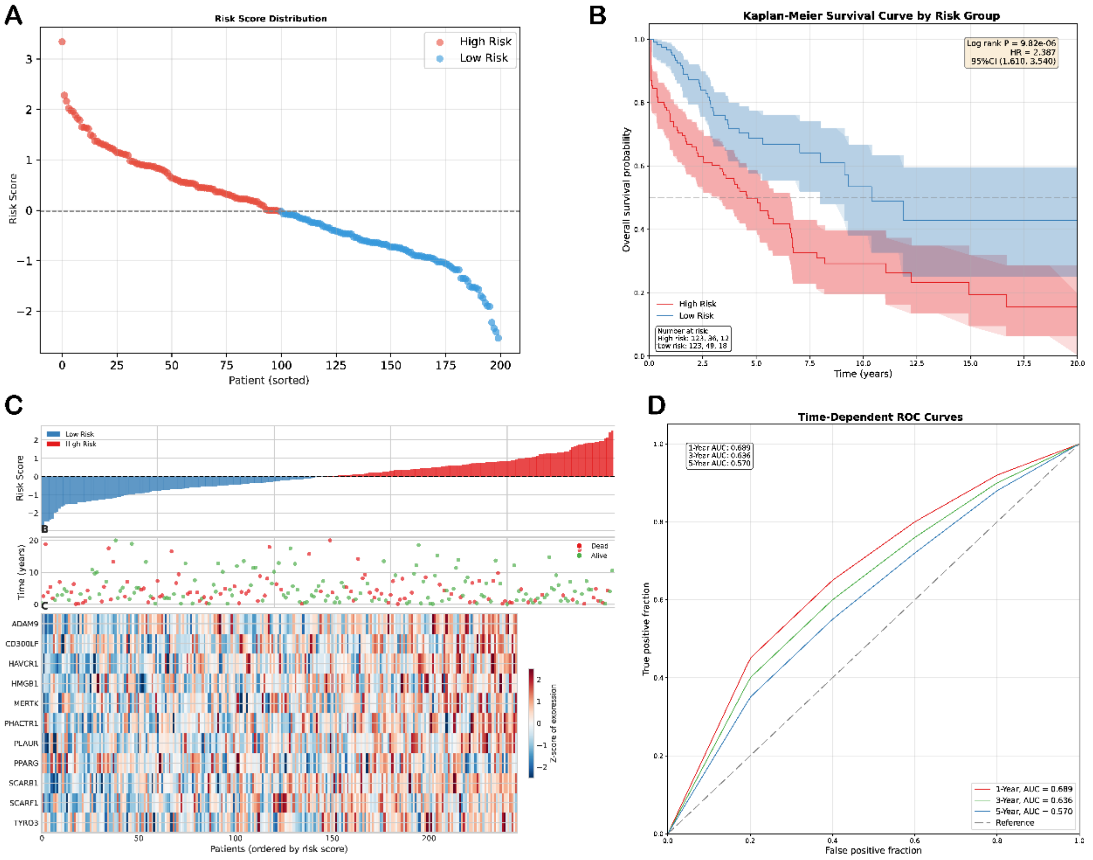

Figure S1. Validation of the 11-gene prognostic signature in the GSE31210 LUAD cohort. (A) Distribution of risk scores and corresponding survival status of patients in the GSE31210 cohort. (B) Kaplan-Meier overall survival curves for high- and low-risk groups in the GSE31210 cohort (HR = 2.387, 95% CI: 1.610-3.540; log-rank P = 9.82×10^{-6}). (C) Expression heatmap of the 11 efferocytosis-related genes in high- and low-risk patients from the GSE31210 cohort. (D) Time-dependent ROC curves for 1-, 3-, and 5-year overall survival prediction in the GSE31210 cohort.

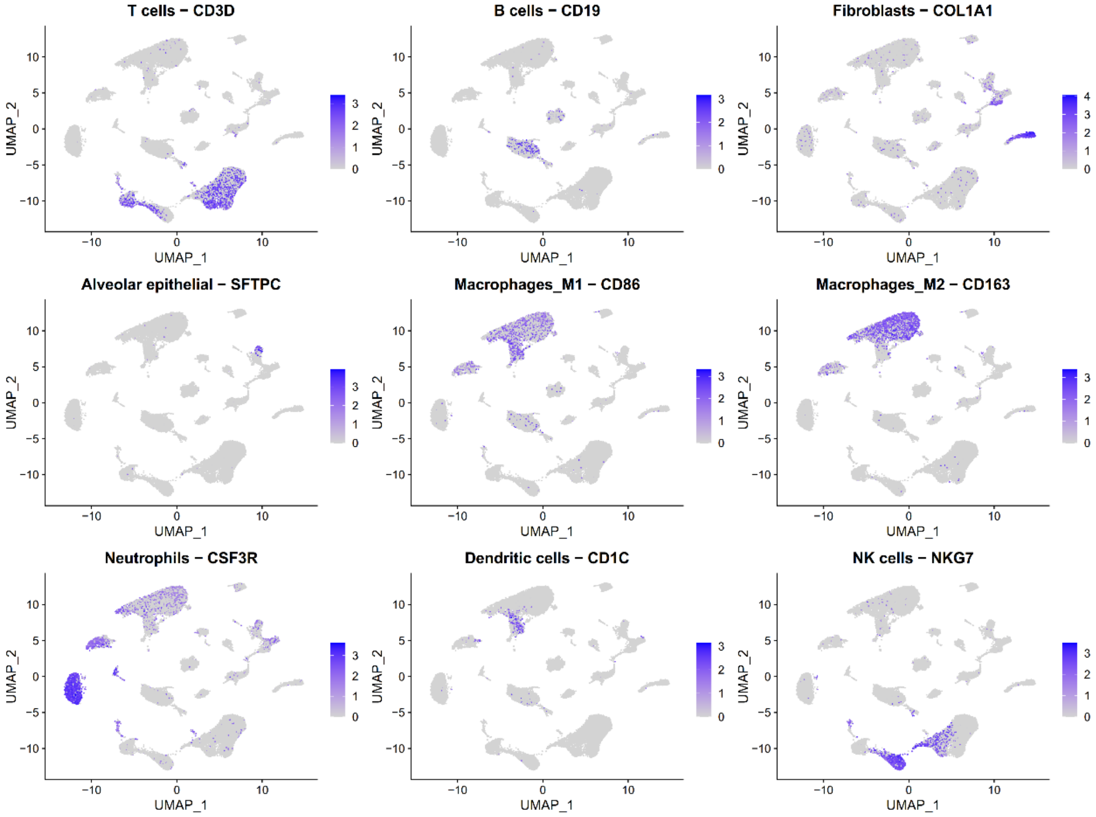

Figure S2. Expression and distribution of canonical marker genes for each cell subpopulation.

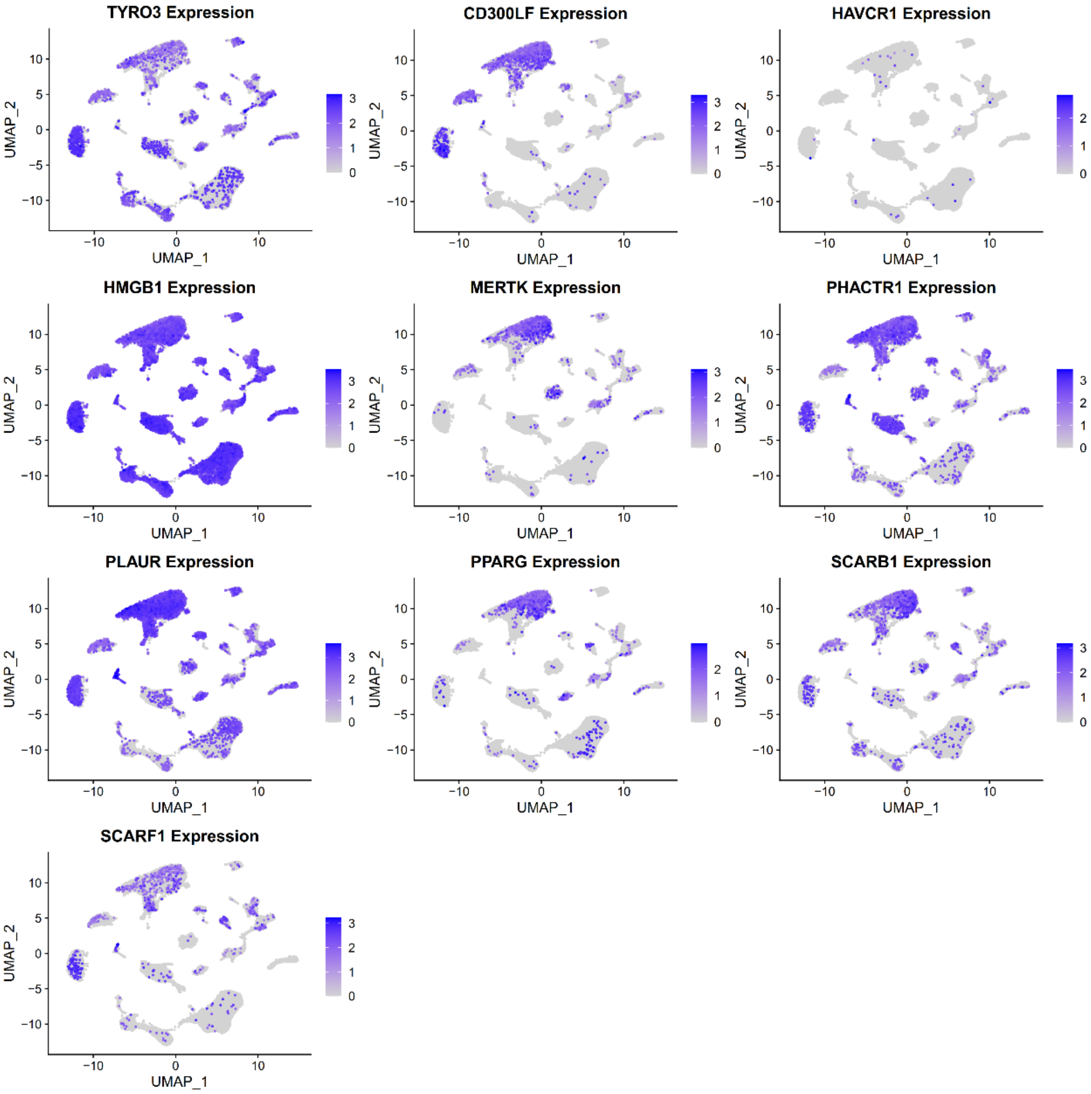

Figure S3. Expression and distribution of key efferocytosis-related genes.

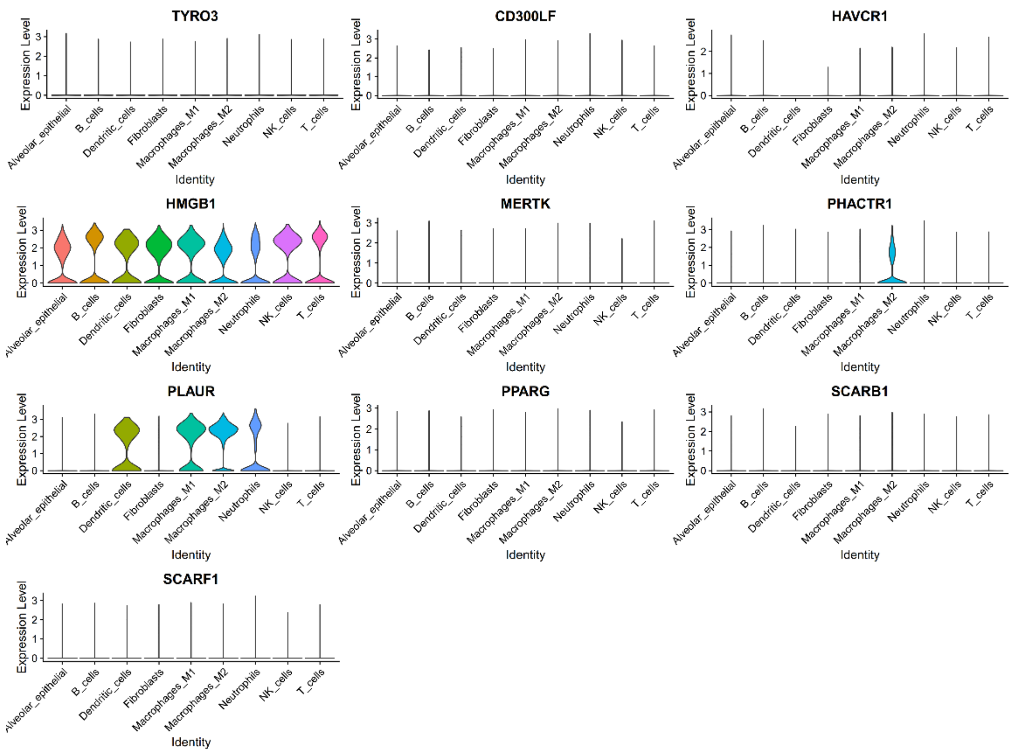

Figure S4. Expression levels of key efferocytosis-related genes across identified cell subpopulations.

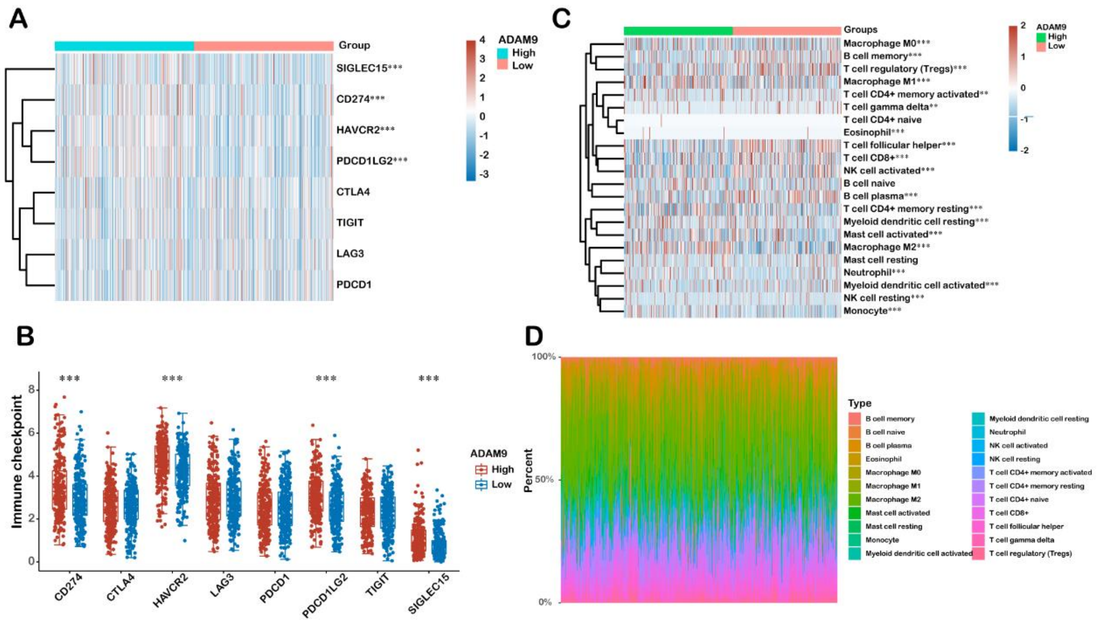

Figure S5. ADAM9 expression and its correlation with immune checkpoints and immune cell infiltration in LUAD. (A, B) Differential expression of immune checkpoints (e.g., SIGLEC15, HAVCR2) between ADAM9-high and -low groups. (C) Immune cell infiltration profiles showing variations between ADAM9-high and -low groups. (D) Proportional composition of immune cell types, illustrating infiltration percentage differences between ADAM9-high and -low groups. ***P* < 0.01, ****P* < 0.001.

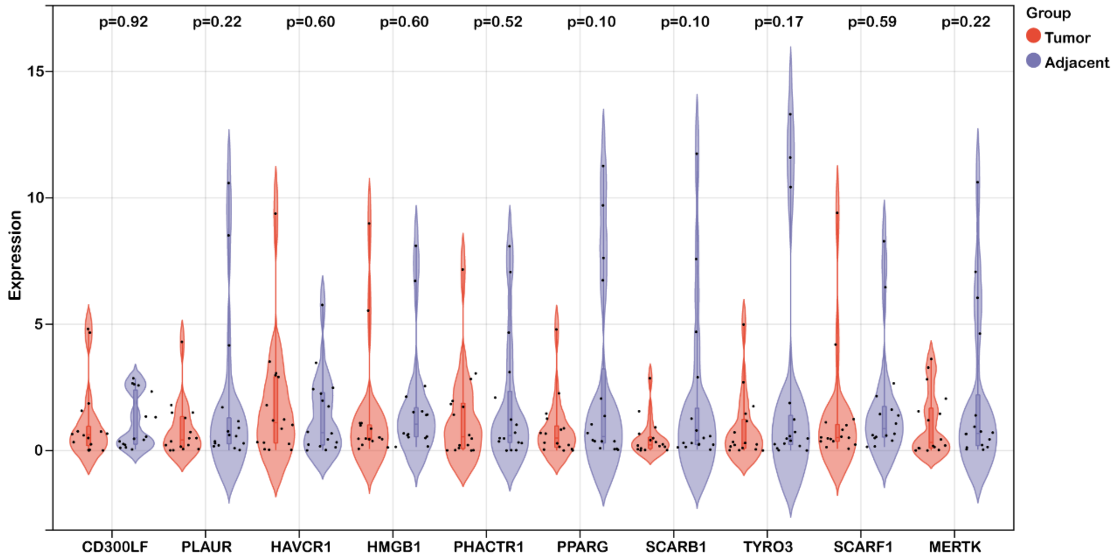

Figure S6. ERG mRNA expression levels were detected via RT-qPCR.

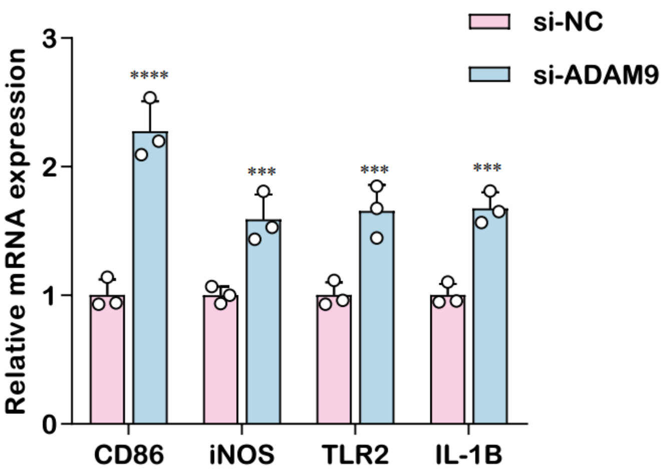

Figure S7. RT-qPCR analysis of M1 polarization markers in si-ADAM9-transfected macrophages. ***$P < 0.001$, ****$P < 0.0001$.

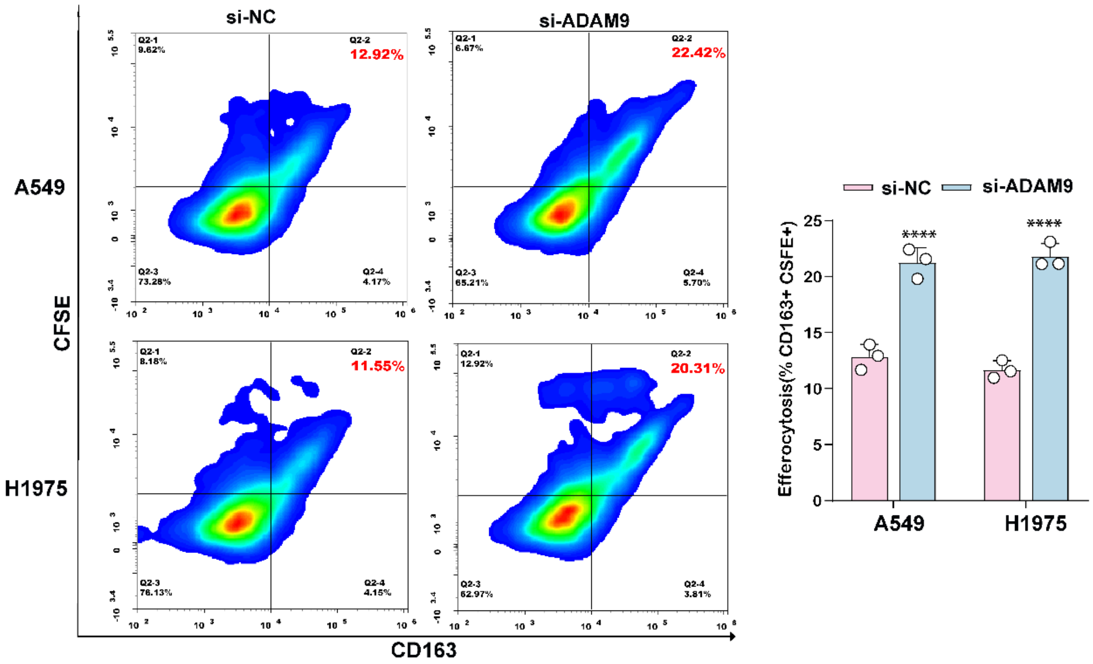

Figure S8. ADAM9 knockdown promotes macrophage efferocytosis of lung cancer cells. Flow cytometry analysis of efferocytosis by $CD163^{+}$ macrophages co-cultured with CFSE-labeled apoptotic A549 and H1975 cells. Macrophages were transfected with si-ADAM9 or si-NC. Representative dot plots and quantification show increased efferocytosis ($CD163^{+}CFSE^{+}$%) in the si-ADAM9 group. ****$P < 0.0001$.
